# Illusory Sensation of Movement Induced by Repetitive Transcranial Magnetic Stimulation

**DOI:** 10.1371/journal.pone.0013301

**Published:** 2010-10-11

**Authors:** Mark Schram Christensen, Jesper Lundbye-Jensen, Michael James Grey, Alexandra Damgaard Vejlby, Bo Belhage, Jens Bo Nielsen

**Affiliations:** 1 Department of Exercise and Sport Sciences, University of Copenhagen, Copenhagen, Denmark; 2 Danish Research Centre for Magnetic Resonance, Copenhagen University Hospital Hvidovre, Hvidovre, Denmark; 3 Department of Neuroscience and Pharmacology, University of Copenhagen, Copenhagen, Denmark; 4 Department of Anaesthesiology, Herlev Hospital, Herlev, Denmark; 5 Department of Anaesthesiology, Bispebjerg Hospital, Copenhagen, Denmark; Macquarie University, Australia

## Abstract

Human movement sense relies on both somatosensory feedback and on knowledge of the motor commands used to produce the movement. We have induced a movement illusion using repetitive transcranial magnetic stimulation over primary motor cortex and dorsal premotor cortex in the absence of limb movement and its associated somatosensory feedback. Afferent and efferent neural signalling was abolished in the arm with ischemic nerve block, and in the leg with spinal nerve block. Movement sensation was assessed following trains of high-frequency repetitive transcranial magnetic stimulation applied over primary motor cortex, dorsal premotor cortex, and a control area (posterior parietal cortex). Magnetic stimulation over primary motor cortex and dorsal premotor cortex produced a movement sensation that was significantly greater than stimulation over the control region. Movement sensation after dorsal premotor cortex stimulation was less affected by sensory and motor deprivation than was primary motor cortex stimulation. We propose that repetitive transcranial magnetic stimulation over dorsal premotor cortex produces a corollary discharge that is perceived as movement.

## Introduction

Sensation of movement in humans relies to a large extent on somatosensory feedback from muscles, joints and skin [1]. However, there is evidence suggesting that efferent signals from motor centers in the central nervous system also provide information that influence sensation of movement [2], [3]. During normal conditions when humans perform movements it is hard to dissociate the relative afferents and efferent contributions to the sensation of movement. In the present study we have identified purely efferent sensations of movement and we suggest that dorsal premotor cortex (PMd) provides corollary discharges [4] that directly give rise to a sensation of movement.

Illusory sensation of movement has been addressed with invasive experiments in which direct electrical stimulation has been used to probe various regions of the brain during surgery. Scarff [5] described the first anecdotal evidence of direct electrical stimulation of premotor cortex that produced a sensation of movement without an apparent movement of the limb. Decades later, stimulation of the supplementary motor area was reported to produce an urge to move [6]. More recently, Desmurget et al. [7] reported that electrical stimulation of the surface of the parietal cortex produced ‘an intention to move’. In contrast to the previous studies, the authors also reported that actual movements caused by stimulation of the premotor cortex were not accompanied by a sensation of movement, despite the fact that the stimulation evoked clear electromyographic activity in the limbs. Therefore, the subjects must also have received sensory feedback from the muscles. All of these direct cortical stimulation studies were performed in patients who had neurological conditions that may have interfered with the normal functioning of cortical networks; and this may explain the discrepancies between their findings. It would therefore be advantageous to study illusory sensation of movement with a non-invasive stimulation technique such as repetitive transcranial magnetic stimulation (rTMS) [8] in healthy subjects who are deprived of sensory feedback. The use of navigated brain stimulation guided by functional magnetic resonance imaging (fMRI) activations makes it is possible to carefully map where the rTMS stimulation causes a sensation of movement without accompanying muscle activity.

Movement sensation in the absence of sensory feedback is thought to be caused by centrally generated motor commands. These motor command copies, known as corollary discharges [4], [9], influence sensory perception and are derived from efference copies [10] using forward models that predict the sensory consequences of a movement. While the computation of the forward model is most likely to take place in the cerebellum [11], [12], the premotor cortex [13]–[15] and supplementary motor area (SMA) [2], [13] have been proposed as likely sites for the creation of the corollary discharge. In particular, the premotor cortex is an interesting candidate because it has been shown to be functionally coupled with the somatosensory cortex in the absence of proprioceptive feedback [15]. The goal of the present study was to test specifically if the PMd or SMA generates the corollary discharge. Furthermore, we tested sensation of movement by stimulating primary motor cortex (M1), which is the primary efferent area of the cortex and posterior parietal cortex (PPC), which has been shown to integrate visual information and motor commands [16].

In order to test if movement can be perceived without sensory feedback and without elicited movement activity, we induced movement and/or sensation of movement with trains of high frequency (20 Hz) rTMS guided by fMRI activations when the sensory and motor neural traffic to and from the limb was blocked using ischemic nerve block (INB) and spinal blockade (SB) in two separate experiments. Using TMS and INB/SB it was possible to perform these experiments in normal healthy volunteers without the side effects of open skull surgery and the possible interactions with disease states that otherwise may interfere with the interpretation of the findings.

## Methods

### Subjects and task

All subjects gave written and informed consent prior to participation. The study (H-A-2008-029) was approved by the local ethics committee of the Capital Region of Denmark (De Videnskabsetiske Komiteer for Region Hovedstaden). Fifteen subjects were recruited for the INB study and ten subjects were recruited for the SB study. The INB experiment was split into a main experiment with ten participating subjects (mean age 25.1y, range 21-38y) and two control experiments where five took part in control experiment 1 (mean age 31.8y, range 23-43y) and four took part in control experiment 2 (mean age 34.8y, range 27-47y) with some overlap between the experiments (see supplementary Table S1). In the SB study only 6 subjects were included in the analysis (mean age 28y, range 25-35y). One subject withdrew from the study during the experiment, one subject received an epidural blockade rather than a spinal blockade, and in two subjects a complete motor block was not obtained.

### Sensorimotor blockade

Afferent and efferent neural transmission was abolished by inflating a tourniquet around the arm to produce a peripheral ischemic nerve block (INB). To abolish neural transmision to the leg, a spinal nerve block (SB) was induced using a subarachnoid injection of bupivacin between L2 and L3 lumbar segments. Thereby two independent measures of sensory and motor blockade were tested in two different body parts, and possible deviations between the two methods could be indentified.

After baseline measurements, the tourniquet in the INB experiment was inflated to ∼250 mmHg. While the subject gradually lost sensation in the forearm and hand, sensation of light touch and passive movement of the fingers was tested using gentle strokes of the skin while subject's eyes were closed. When the subjects had lost skin sensation, which typically occurred 20–25 min after initiation of ischemia, the subjects were asked to perform voluntary finger and hand movements. Subjects on average lost the ability to perform voluntary finger/hand movements ∼27–30 min after the tourniquet was inflated. TMS trains were then applied over the three motor and the control regions with approximately 6 trains over each area in order to test sensation without actual movement. The experiment during INB was kept short, always lasting less than 10 min, in order not to induce too long a period of discomfort for the subjects. This put a limit to the number of pulse trains that were applied to the subjects during INB. However, if placement of the coil in accordance with the targets over the different regions were made quickly, we tried to obtain as many trains as possible during the 10 min.

In the SB experiment the subjects had been fasting minimum six hours prior to the procedure. An intra venous access was secured in the left forearm during the whole procedure. A pulse oximeter, blood pressure cuff and electrocardiograph were placed for baseline measurements. Heart rate and arterial oxygen were monitored continuously, and blood pressure was monitored at five-minute intervals the first 30 minutes, and thereafter every fifteen minutes. With the patient in sitting position the gap between L2 – L3 was identified and standard surgical disinfection and sterile procedure was followed throughout the intratechal injection. With a 27-gauge pencil-point needle, bupivacin (“Macain High Density”) was deposited into the subarachnoid space in a midline approach. The amount of bupivacain was varying between 2.5–3.0 ml according to the subject's height. Immediately after drug injection the subjects were placed in supine position with the headrest of the bed elevated 10–15 degrees.

Sensory block was assessed bilaterally by loss of cold sensation. Total sensory block was observed after fifteen minutes in all of the included subjects, with a thermo boarder at the L2 –L3 level. The degree of motor block was determined according to the Bromage scale [17] (1 = free movements of the legs and feet ∼0% block; 2 = just able to flex knees with free movement of feet ∼33% block; 3 = unable to flex knees, but with free movement of feet ∼66% block; 4 = unable to move legs or feet ∼100% block. After approximately 45 minuets all included subjects had a score of 4 on the Bromage scale. After the TMS experiments the motor block of the right leg was assessed again and for all subjects the score was still 4. All subjects were observed until they had complete recovery of motor function of both legs, and bladder control.

### TMS experiment

**Figure 1:**
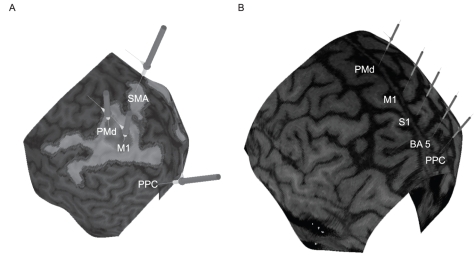
Areas stimulated with rTMS. A shows the regions (from a single subject) that were stimulated in the experiment using INB of the arm. The sites are shown on a peeled cortex surface with the functional activation maps overlayed onto the cortex in the INB experiments. The sticks indicate the approximate centre of the regions M1, PMd, SMA and PP, where the centre of the TMS coil was placeed, however the extent of the stimulation of each region depends on the spread of the magnetic field induced by the coil. B shows the regions stimulated (in a single subject) in the experiment using SB of the lower limbs. The sites are shown on a standard brain, i.e. not the subject's own brain.

In the INB experiment TMS was applied over the three motor regions (M1, PMd, SMA) in order to induce sensation of movement. Stimuli were applied at 20 Hz in pulse trains lasting 500 ms (i.e. 11 pulses). In addition, stimulation over the posterior part of the parietal cortex served as a control region (PPC) (See Figure 1A). The region was placed as far posterior as possible within the range of the navigation camera. Stimulation intensity was set to 100% of resting motor threshold (rMT). If the subject did not report a clear sensation of movement at 100% rMT, the stimulation intensity was increased slightly to 105–110% rMT, this was the case in 7 of the 10 subjects in the main INB experiment. In control experiment 2 (described in the supplementary Text S1's Supplementary control experiments section) we also applied trains of stimuli for 150 ms (i.e. 4 pulses). During all measurements the subjects were instructed to remain relaxed and keep their eyes shut. EMG was recorded from the 6/4 (INB/SB) different muscles from 300 ms before the first stimulation and for a total of 2000 ms for each stimulation train. The preceding MRI procedures are described in the supplementary Text S1's Supplementary methods section.

In the SB experiment TMS was applied over five different regions. The motor hotspot (M1) for evoking MEPs in SOL and TA was first identified and the rMT was determined (similar approach as for the INB experiment). A dorsal premotor region (PMd) was defined from the location of the M1 hotspot approximately 1.8–2.0 cm anterior to M1. Additionally a primary sensory region (S1) was defined located 1.8–2.0 cm posterior to M1, and an even more posterior regions (BA 5), further 1.8–2.0 cm back. Finally, a posterior parietal region (PPC) was located ∼2 cm further back (See Figure 1B). Since MRI scans were not obtained, the location of the stimuli could not be located accurately based on brain anatomy or functionally activated areas. Therefore, the coverage from anterior premotor regions to posterior parietal regions was more extensive compared with the INB experiment, and therefore also include intermediate sensory areas. However, the accuracy and stability of coil placement was ensured using BrainSight and a normalised brain.

In all experiments TMS was applied using a Magstim 200 Rapid stimulator (with four booster modules) (The Magstim Company, Whitland, Dyfed, UK) and a custom-made batwing figure-of-eight shaped coil controlled from a Micro 1401 Mk II and Signal 4.00 software (Cambridge Electronic Design, Cambridge, UK.).

### Subjective assessment of movement sensation

Baseline experiments were performed before induction of ischemic nerve block (INB) and spinal blockade (SB). The TMS coil was placed over M1 and a train of 11 TMS pulses was applied. The subject's task was to remember the sensation of movement evoked by this stimulus train and score subsequent sensation in relation to this sensation. This served as a baseline reference corresponding to the strongest sensation of movement experienced during the experiment. This sensation was rated as 5 on a scale from 0–5 where 0 corresponded to no sensation of movement and 5 as the strongest sensation of movement. 1 corresponded to a very light sensation of movement barely perceptible by the subject. The intermediate points on the scale were subjectively decided upon by the subject such that they matched a linear increase of sensation of movement, i.e. 1 corresponded to approximately 20% of the strongest possible movement, 2 to 40% etc. During every trial the subjects had their eyes closed in order not to see whether they moved or not.

In the INB experiment subjects were placed comfortably in a seat with their right arm resting on an armrest. Ag/AgCl electromyography (EMG) bipolar electrodes (Amba, Skovlunde, Denmark) were placed with interelectrode distance of 2 cm at the first dorsal interosseus (FDI), abductor digiti minimi (ADM), brachioradialis (BR), extensor carpi radialis longus (ECR), flexor carpi radialis (FCR), and at the biceps brachii (BB) muscles. The electrodes were connected to wireless EMG preamplifiers (x1000) (ZeroWire, Aurion, Milano, Italy), bandwidth was 10–1000 Hz. EMG signals were recorded at 2000 Hz using a Micro 1401 Mk II for AD-conversion and Signal 4.00 software (Cambridge Electronic Design, Cambridge, UK). A tourniquet was placed around the upper arm of the subject. The tourniquet and BB electrodes were placed such that the tourniquet did not cover the electrodes.

In the SB experiment subjects were lying comfortably in a bed. EMG electrodes were placed over soleus (SOL), tibialis anterior (TA), biceps femoris (BF), and rectus femoris (RF). Cables were attached to the electrodes and the EMG signals were amplified (x1000) using custom-made amplifier modules, the signals were high pass (25 Hz) and low pass (1000 Hz) filtered and recorded at 2000 Hz using Signal 4.00 software and a Micro 1401 Mk II (Cambridge Electronic Design, Cambridge, UK).

### Statistics

Repeated measures ANOVA were used to test the effect of sensory nerve block (INB and SB) and stimulation site (M1, PMd, SMA, PPC in the INB experiment; M1, PMd, S1, BA5, PPC in the SB experiment) and the interaction (block × site) on the induced sensation of movement. The data were not normally distributed, however the normality tests failed due to extremes in the data. Nevertheless we have chosen to use the parametric ANOVA test because it is not very sensitive to moderate deviations from normality [18], [19]. This test permits a more straightforward analysis of interaction effects, which is the main focus of the study. Post hoc tests were made for the pair-wise comparisons (regions: before vs. during block) with Bonferroni correction for multiple comparisons using the total number of relevant comparisons, i.e. not including comparisons of two different sites between present or absent sensory nerve block, such as comparing e.g. PMd before with SMA during INB. In the cases where a stimulated region was compared with the PPC control region Dunnett's two sided multiple comparison test with control was used.

Linear regression analysis was performed in order to test the correlation between the number of evoked MEPs after each stimulation with the subjectively reported sensation of movement.

## Results

### Motor evoked potentials and perceived movements

**Figure 2:**
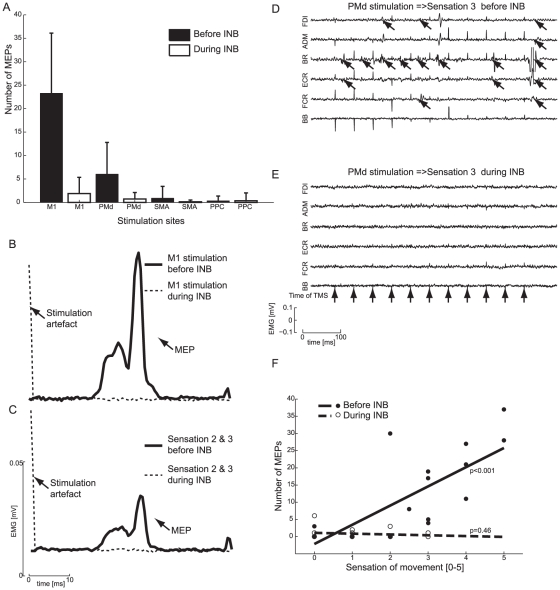
Motor evoked potentials and sensation of movement in INB experiment. A shows the average number of evoked MEPs in the INB experiment before INB (in black) and during INB (white) (error bars indicate standard deviation). B shows a MEP (EMG rectified) from muscle BR average across six stimulation trains over M1 (one subject) before INB (black) and during INB (dashed). No MEP is seen during INB. C shows a MEP (EMG rectified) from muscle BR average across all stimulation trains giving rise to a sensation corresponding to 2 or 3 (one subject) before INB (black) and during INB (dashed). No MEP is seen during INB. In both cases the sensation of movement is the same but during INB there is no accompanying muscle activity. D, E shows a typical pattern of muscles activities (one subject) before INB (D) and during (E) (PMd stimulation giving rise to a sensation of movement = 3) after a train of TMS (see the arrows in the bottom of panel E indicating when the individual pulses were given), the same sensation of movement is present in both cases, one with and one without any accompanying MEPs (indicated by angled arrows). F shows the total number of MEPs in six muscles after each stimulation train as a function of the sensation of movement before INB (large dots, full line) and during INB (open circles, dashed line). The regression lines and p-values are plotted as well. Example is taken from a single subject.

**Figure 3:**
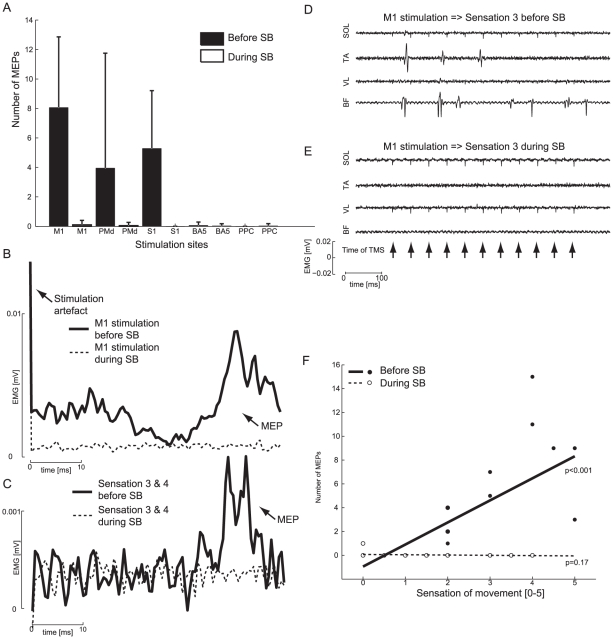
Motor evoked potentials and sensation of movement in SB experiment. A shows the average number of evoked MEPs in the SB experiment before SB (in black) and during INB (white) (error bars indicate standard deviation). B shows a MEP (EMG rectified) from muscle TA average across six stimulation trains over M1 (one subject) before SB (black) and during SB (dashed). No MEP is seen during SB. C shows a MEP (EMG rectified) from muscle TA average across all stimulation trains giving rise to a sensation corresponding to 3 or 4 (one subject) before SB (black) and during SB (dashed). No MEP is seen during SB. In both cases the sensation of movement is the same but during INB there is no accompanying muscle activity. D, E shows a typical pattern of muscles activities (one subject) before SB (D) and during (E) (M1 stimulation giving rise to a sensation of movement = 3) after a train of TMS (see the arrows in the bottom of panel E indicating when the individual pulses were given), the same sensation of movement is present in both cases, one with and one without any accompanying MEPs. F shows the total number of MEPs in four muscles after each stimulation train as a function of the sensation of movement before SB (large dots, full line) and during SB (open circles, dashed line). The regression lines and p-values are plotted as well. Example is taken from a single subject.

Loss of sensation during INB and SB was tested before the second half of the experiment using skin touch and passive movements of the limbs as described previously. As shown in Figure 2A and 3A motor blockade was also present during INB and SB where very few MEPs were elicited by the TMS compared to before INB and SB. An average of the evoked EMG after all TMS pulses from a representative subject clearly shows that MEPs were suppressed during INB and SB compared with normal sensory feedback (see Figure 2B/3B and 2C/3C ). It is also evident that during two trials where the sensation of movement was the same before and during INB or SB, no MEPs were elicited during INB or SB (se Figure 2D/3D and 2E/3E).

In order to test the reliability of the subjective movement sensation scale, the total number of TMS-evoked muscle twitches was correlated with the subjective sensation of movements. There was a significant correlation (p<0.001) between the movement sensation score and the number of TMS-evoked muscle twitches for all subjects before INB was induced, i.e. when normal afferent and efferent nerve conduction was present. However, during INB only one of the subjects showed a significant correlation (p<0.001). For the SB experiment all subjects also showed a significant correlation (p<0.001) between the number of MEPs and sensation of movement before SB, whereas during SB none of the subjects showed a significant correlation (p>0.001), one subject showed a tendency (p = 0.008) and one subject was excluded from analysis due to an amplifier-failure.

As shown in Figure 2F/3F some stimulation trains did give rise to a few MEPs even though the experiment was carried out as late as 30–40 min after INB was introduced. However, the pattern of muscle activity evoked by TMS looked similar to what is shown in Figure 2D/3D during normal sensory conditions before INB and as in Figure 2E/3E during INB in all subjects.

### Sensation of movement during ischemic nerve block

**Figure 4:**
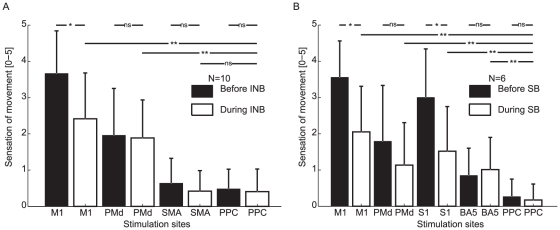
Sensation of movement induced by rTMS. A shows the mean sensation (error bars indicate standard deviation) of movement evoked over the four regions before INB (black) and during INB (white) from the main experiment using INB of the arm. B shows the mean sensation (error bars indicate standard deviation) of movement evoked over the five regions before SB (black) and during SB (white) from the experiment using SB of the lower limbs. Significant differences are indicated with * when p<0.05 using Bonferroni correction for multiple comparisons and with ** when p<0.05 using Dunnett's two sided multiple comparison test with control.

The subjective sensation of movements from the INB experiment are presented in Figure 4A. Clear sensations of movement were present before INB when M1 and PMd were stimulated (significantly different from PPC stimulation (p<0.05, Dunnett's two sided multiple comparison test with control)). SMA stimulation did not evoke significant difference in sensation of movement compared with PPC.

In the INB experiment ranked sensation of movement was entered into a repeated measures ANOVA with the stimulation sites (M1, PMd, SMA and PPC) and sensory block (absent or present) as within subject factors. This analysis revealed a significant block × site interaction (p<0.001, F_3,9_ = 8.42). Site showed significant effects on sensation of movement (p<0.001, F_3,9_ = 58.64) and, finally block as significant effect on sensation of movement (p<0.05, F_1,9_ = 7.77).

Post hoc tests reveal that M1 induced sensation of movement is the only site significantly affected by INB (p<0.05, Bonferroni corrected for multiple comparisons). During INB PMd and M1 induced sensation of movement is significantly different from the control region PPC. PMd induced sensation of movement is not affected by INB (see Figure 4A).

### Sensation of movement during spinal anaesthesia

The subjective sensations of movements from the SB experiment are presented in Figure 4B. Clear sensations of movement were present before SB when M1, S1 and PMd were stimulated (significantly different from PPC stimulation (p<0.05, Dunnett's two sided multiple comparison test with control)). BA5 stimulation did not evoke significant difference in sensation of movement compared with PPC before SB.

In the SB experiment ranked sensation of movement was entered into a repeated measures ANOVA with the stimulation sites (M1, PMd, S1, BA5 and PPC) and sensory block (absent or present) as within subject factors. This analysis revealed a significant block × site interaction (p<0.01, F_4,5_ = 5.41), Site showed a significant effect on sensation of movement (p<0.001, F_4,5_ = 12.86) and sensory block showed a trend on sensation of movement (p<0.06, F_1,5_ = 5.10).

Post hoc tests reveal that M1 and S1 induced sensations of movement are significantly (p<0.05, Bonferroni Multiple comparison test) affected by SB. During SB M1, S1, PMd and BA5 induced sensation of movements are significantly different from the control region PPC (See Figure 4B).

## Discussion

The results show that rTMS induces a sensation of movement in the absence of sensory feedback when applied over M1 and PMd. A sensory and motor block was accomplished using INB and SB and the effect was present in both the arm and the lower limbs. The sensation of movement was not accompanied by movement and may therefore be characterized as a movement illusion. The illusion was present in the arm when with M1 and PMd stimulation, and additionally in somatosensory areas of the leg when the leg area of the cortex was stimulated with rTMS. Interestingly, the absence of sensory feedback did not affect the induced sensation of movement over PMd to the same extent as that for the movement illusion evoked by M1 stimulation.

At baseline, with normal sensory feedback, the movement illusion was not as strong for PMd stimulation compared with M1 stimulation. Therefore, it could be argued that the lack of change in the sensation evoked by the PMd stimulation could be explained by a lower sensitivity to the effect of INB for the weaker sensation. However, when the M1 stimulation intensity was reduced in control experiment 1 (see supplementary Text S1's Supplementary control experiment section and supplementary Figure S1) such that the induced sensation was similar to the sensation of movement induced over PMd, we found that even intermediate (subjective scale ∼2–3) sensations of movements were sensitive to INB when induced over M1. The lack of change in movement sensation associated with PMd stimulation suggests that PMd has a different role in movement sensation than does M1. A study in monkeys showed that premotor cortex activity represented the perception of movement and not the actual movement [20]. Theoretical work [13] has suggested that efference copies are generated in the premotor cortex and that efference copies may be important for perception. We propose that the findings of the present study reflect the notion that PMd generates a corollary discharge when stimulated with TMS, and that this directly influences perceptual areas including somatosensory cortex and possibly also M1. The network of PMd, M1 and possibly also S1 [15] is responsible for generating a sensation of movement. Ellaway et al. [14] showed that M1 did not produce such a corollary discharge but suggested that premotor cortex might, although the study did not test this possibility directly. We have shown that stimulation induced sensation of movement over PMd is less sensitive to sensory and motor deprivation than is M1, and as the results in control experiment 1 shows, it is not due to less sensitive subjective measures of movement perception for low intensity movements.

It has been suggested that the mirror neuron circuitry linking areas of the ventral premotor cortex with the parietal area PF and the superior temporal sulcus generates the necessary components in establishing inverse models, whereby observed actions are coded into motor plans, and forward models that predicts the sensory (visual) outcome of the movement [21]. In the present study we did not engage our subjects in tasks where they were supposed to view their own limbs moving (with and without sensorimotor deprivation) so we cannot provide evidence of whether we in fact targeted mirror neurons, because our tasks were not specifically designed to target that question. However, we may have engaged similar networks, in particular the premotor stimulation, may have engaged mirror neuron related networks.

Haggard and Whitford [2] showed that TMS stimulation over SMA prior to a voluntary movement removed the sensory suppression effect, which normally accompanies voluntary movements [22]. Electrophysiological [23]–[25] and neuroimaging [26] evidence shows that SMA is involved in early preparatory components of voluntary movements, where one function may be to predict the sensory consequences of the movements as indicated by Haggard and Whitford [2]. Using similar stimulation intensity over SMA, i.e. approximately resting motor threshold for evoking MEP after stimulation over M1, we were not able to induce a sensation of movement. This suggests that the central cancellation of predicted sensory feedback is not directly related to the sensation of movements although both mechanisms require top down modulation of sensory areas. This likely explains our negative finding of absent sensation of movement after rTMS over SMA. However, direct cortical stimulation of SMA has previously induced movements [6], so an alternative explanation could be that we were not able to efficiently stimulate SMA with TMS when the TMS was not accompanied by a voluntary effort, as was the case for the study by Haggard and Whitford [2].

The stimulation trains over PPC did not evoke any sensation of movement. We chose this location based on our previous observation that areas in the posterior medial part of the parietal lobule showed properties related to integration of visual feedback and motor commands [16]. By choosing this area we could explore whether stimulation of the region would lead to induced sensations of movements, but this was not the case. Therefore the stimulation over PPC was used as a control for comparison to stimulation over the other regions. A recent study by Desmurget et al. [7] showed that stimulation directly on the cortical surface of parietal areas gave rise to “an urge to move”. In three of the four patients that were stimulated, the areas that were stimulated, were located more lateral than any of the areas that was used as PPC in the present study. In one of the patients the location of Desmurget et al's stimulation site was close to where we stimulated, but the brain tumour that this patient suffered from was also located nearby and could have induced cortical reorganisation that may turn out to produce different results than what would have been found in healthy subjects. Finally, Desmurget et al. used 4 s stimulation trains where we only produced sensations of movement over M1 and PMd with 500 ms trains. These differences in location, stimulation and the fact that Desmurget et al. studied “an intention to move” rather than “a sensation of movement”, may explain why we did not observe induced sensations of movement when PPC was stimulated. Finally, the difference between direct cortical stimulation during surgery and the application of high frequency rTMS may be so different, and therefore give rise to the differences between our study and the study of Desmurget et al. [7] and the findings of Fried et al. [6]. One difference is also that direct cortical stimulation often is conducted using much higher frequencies [6], [7]. Therefore further studies are needed in order to clarify the functional differences between direct cortical stimulation and rTMS.

One explanation could be that rTMS induces more widespread activation due to the extent of the magnetic field and accompanied stimulation area compared with small surface electrodes used during surgery. Another explanation would be that the stimulation of motor areas creates feedback activation of somatosensory cortex, whereas direct somatosensory stimulation requires longer stimulation times in order to create reverberating circuits with other regions.

One surprising observation in the Desmurget et al. [7] study is that their stimulation-evoked movements failed to produce any movement sensation despite the fact that the movement itself must have evoked some afferent traffic. In contrast, the present study reveals a significant correlation between the motor output (number of MEPs in the arm muscles) and the subjective sensation of the movement. This raises the possibility that the patients investigated by Desmurget et al. may have been severely affected by their tumours or, possibly, influenced by sedatives, although the latter is not described in the paper. Their finding is also very surprising, because awake subjects easily can detect single MEPs evoked by TMS, either due to a conscious percept of the motor command or the sensory feedback coming from the muscle twitch.

In the INB and SB experiment stimulation trains lasted 500 ms. The duration was inspired by the minimal duration of stimulation trains used by Libet et al. [27] in order to produce perceivable sensory sensations. Stimulation trains lasting 500 ms or more evoked sensory sensations whereas shorter duration stimulation trains did not. However, in the second control experiment (described in the supplementary Text S1's Supplementary control experiments section) we also found that shorter (150 ms) magnetic stimulation trains produced perceivable movements with and without actual movement (see supplementary Figure S2). This further suggests that there is a qualitative difference between direct electrical cortical stimulation and rTMS as a mean to produce illusions of movement.

Changes in cortical representation of body parts affected by sensorimotor deprivation have been studied with PET [28] showing increased cerebral blood flow during INB but only during rest. MEP amplitudes in muscles proximal to INB have also been studies with TMS [29], revealing that these were increased in a proximal muscle during INB. However, in the present study the movement sensation reported by subjects was constricted to finger and wrist movement for the INB experiment and ankle joint rotation for the SB experiment. Only one subject showed elevated levels of MEP in the biceps muscles proximal to the tourniquet.

We have shown that in the absence of sensory feedback and accompanying movements, caused by ischemic nerve block or spinal block, it is possible to induce a movement illusion using rTMS over M1 and PMd for both the arm and the lower limbs. We have shown that the sensation of movement evoked by rTMS when applied over PMd is not affected by the absence of sensory feedback, to the same degree as when applied over M1, suggesting that a corollary discharge evoked by TMS over PMd induces sensation of movement.

## Supporting Information

Text S1 Supplementary methods section and supplementary control experiments section.
(0.04 MB DOC)

Figure S1 Figure S1 shows the mean sensation of movement (error bars indicate standard deviation) with high and low intensity TMS before INB (black) and during INB (white).
(0.32 MB EPS)

Figure S2 Figure S2 shows the mean sensation of movement (error bars indicate standard deviation) with long (500 ms) and short (150 ms) stimulation trains before (black) and during INB (white).
(0.34 MB EPS)

Table S1 Overview of subject's participation. Age and gender of each subject is written in parenthesis. Subjects marked with X participated in the study. Subject marked with * were lab members and had prior knowledge of the purpose of the experiment, whereas the subjects participating in the main experiment were completely naive towards the hypotheses tested in the experiment. For the SB experiment, subjects marked with ∅ were not included in the analysis (see Methods for details).
(0.05 MB DOC)
