# Supplementary material for: Illusory Sensation of Movement Induced by Repetitive Transcranial Magnetic Stimulation
Source: PLoS One. 2010 Oct 11;5(10):e13301. doi: 10.1371/journal.pone.0013301 (PMC2952623; doi:10.1371/journal.pone.0013301)
Supplement: Text S1 — Supplementary methods section and supplementary control experiments section. (0.04 MB DOC) [file pone.0013301.s001.doc]

**Supplementary methods**

***Magnetic Resonance (MR) scanning***

Prior to the TMS experiment with INB the subjects were MR-scanned in order to obtain structural and functional MR-scans used for navigated TMS stimulation

***MR-scanning and analysis procedures***

All scans were acquired using a 3 Tesla MR scanner (Siemens Magnetom Trio, Erlangen, Germany) using an 8-channel head coil. For functional scans, a whole brain gradient-echo echo-planar imaging (EPI) sequence with a repetition time (TR) = 2400 ms, echo time (TE) = 30 ms, and flip angle = 90^o^ was employed, using a 64 x 64 matrix with an in-plane resolution of 3 x 3 mm^2^. Each volume consisted of 42 slices, with slice-thickness 3 mm. The slices were obtained in an interleaved fashion beginning with the bottom slice. Each functional experiment consisted of 240 EPI volumes, lasting 600 s in all. One or two sessions of fMRI scan were obtained in each subject. Structural scans were also acquired using a magnetisation prepared rapid gradient echo (MPRAGE) sequence with TR = 1540 ms, TE = 3.93 ms, flip angle = 9^o^ and 192 x 256 x 256 acquired matrix, with a resolution of 1 x 1 x 1 mm^3^. The subjects’ pulse and respiration were recorded using an MR-compatible pulse oximeter, and a respiration belt, and sampled at 50 Hz.

***Task during MR***

During the sessions of fMRI scan subject performed two types of finger movements with their right hand: a pre-learned sequence of button presses using digits 2-4-3-5 or visually guided random sequences of similar button presses. Both types of movements were paced at a frequency of 1 Hz.

Visual guidance was similar in the two tasks; a gray hand was displayed on a black background. For the pre-learned sequence a dot was flashing in the middle of the hand at the pace of 1 Hz, where as in the visually guided task, the dot was flashing below the finger (digit 2-5) subjects should move. The two types of movements were performed in blocks of 15 s with 15 s of rest between each block.

***MR-data analysis***

The EPI time series were realigned to the mean image of all the EPI images and unwarped in order to account for deformations caused by the non-linearities of the fast gradients. The MPRAGE image was also unwarped. Then the EPI images were realigned to the MPRAGE and re-sliced to the mean EPI images. Finally a 6 mm isotropic Gaussian smoothing kernel was applied to the EPI images.

Statistical analysis of the EPI time series was performed as a general linear model. Two regressors, corresponding to the on-off periods of the pre-learned and visually guided finger movements, were modelled as boxcar functions and convolved with a hemodynamic response function.

In order to correct for the structured noise induced by respiration and cardiac pulsation we included RETROICOR (RETROspective Image based CORrection method) nuisance covariates in the design matrix (Glover et al., 2000;Lund et al., 2006). Movements parameters were also included in the analysis, which accounted for spin history effects as well (Friston et al., 1996). A high pass filter with a cut-off frequency at 1/128 Hz was also applied.

Thresholded (p<0.05, corrected for multiple comparison) SPM{t}s were obtained for the conjunction of the two movement types. In all subjects these maps consistently provided activation of primary motor regions (M1), dorsal premotor regions (PMd) and supplementary motor regions (SMA) and were used for navigated brain stimulation in the TMS-part of the experiment.

***Navigated brain stimulation***

BrainSight 1.7 (Rogue Research software, Montreal, Quebec, Canada) was used for MR-navigated TMS using the structural and functional images obtained from the MRI scan for the INB experiment. The TMS coil was placed over the motor area (M1) identified from the functional scan and further determined from a mapping procedure in order to optimally elicit MEPs in the forearm and hand muscles, the area will afterwards be named hotspot. The threshold for producing motor evoked potentials in the arm muscles by stimuli applied over the hotspot was determined at rest (rMT) in each subject (3 out of 5 consecutive stimulations produced an MEP larger than 50 μV in one or more of the muscles). For the SB experiment MRIs could not be obtained and a standard brain was therefore used to position the coil with BrainSight instead.

**Supplementary control experiments**

Two control experiments were also performed: In control experiment 1 it was tested whether subjects were able to differentiate the subjective sensation of movement between high and low intensity TMS over one area (M1) when feedback was blocked by INB.

In control experiment 2 it was tested whether both long duration (500 ms) and short duration (150 ms) trains of TMS induced a sensation of movement during INB, and whether long and short trains were equally affected by INB. Libet et al (1964) showed that only long (≥500 ms) stimulations gave rise to perceivable sensory stimulation, so here we tested whether the long stimulation trains used in the main experiment were qualitatively different from short stimulation trains.

In control experiment 1, subjects also received low intensity stimulations over M1. Here the intensity of the stimulation was adjusted so that the induced sensation of movements was approximately at the same level as the sensation induced when PMd was stimulated at full intensity. This level usually corresponded to 100% rMT because subjects did not feel a strong sensation of movement before stimulating at 105-110% rMT. This experiment was carried out in order to test whether a baseline sensation that was lower than the sensation evoked by the M1 stimulation, was as sensitive to INB as the high intensity stimulation. INB was administered in the same way as in the main experiment.

In control experiment 2, subjects received short (150 ms) and long (500 ms) trains of TMS over M1, PMd and PPC in order to compare the relation between movement sensation and stimulation duration with and without sensory feedback. INB was administered in the same way as in the main experiment.

In control experiment 1 high and low intensity M1 stimulation was compared. This was done because the PMd stimulation in the main experiment with normal sensory feedback gave rise to a weaker sensation of movement than the M1 stimulation. We were therefore concerned whether this lower initial sensation of movement was less affected by INB than the higher levels of sensation. However, we found that the sensation of movement produced by both high intensity (p=0.0076, one-tailed paired t-test) and low intensity (p=0.028, one-tailed paired t-test) M1 stimulation was significantly affected by INB. Furthermore, the interaction (Intensity times intervention) was not significant, although there was a statistical trend (p=0.058). Finally, the main question addressed here was whether a low baseline sensation of movement could be affected by INB, and this was the case. This finding is opposite to what was the case for the PMd stimulation, where the sensation was not affected by INB (See Figure S1).

In control experiment 2 long (500 ms) and short (150 ms) duration trains were compared. The motivation for performing this test was to see whether the findings of Libet et al (1964), in which it was found that only long stimulation trains (>500 ms) were perceivable, also applied for this specific type of stimulation. We found that sensation of movement was significantly affected by site (p<0.001, F=72), duration (p=0.001, F=12.71) and INB (p=0.0015, F=11.85), there was a significant site-by-INB interaction (p=0.0006, F=9.27), however none of the other interactions were significant, i.e. site-by-duration (p=0.1502, F=2), INB-by-duration (p=0.9146, F=0.01), site-by-duration-by-INB (p=0.979, F=0.02) (See Figure S2).

**Reference stimulation**

Although the reference stimulation was elicited over M1 corresponding to a clear sensation of movement = 5, it is evident from the data, that the mean sensation of movement with sensory feedback across all subjects is slightly lower ~ 3.5. One explanation for this discrepancy may be that subject’s attention is directed more towards the first reference stimulation because they are told to remember exactly this evoked sensation of movement as the reference. This enhanced attention towards the reference stimulation may also in itself enhance the effect of the stimulation, giving rise to increased motor output. However, it is unlikely that this effect explain any of the observed findings. In control experiment 2 the sensation of movement is higher for the long stimulation trains than what is found in the other experiments. Although subjects were asked to rate the strongest sensation with long and short trains as 5 in both cases, they may have had difficulties when two qualitatively trains were judged interleaved during the experiment. Hence, we believe this is the cause of the difference in average sensation for the long trains compared with the other experiments.

The choice of using 20 Hz trains was made mainly because there is increased corticomuscular coherence (EEG-EMG) in the beta-band during tonic contractions (Conway et al., 1995). Ideally we would have liked to use higher frequencies in order to mimic phasic movement, but we were limited by TMS hardware constraints, as the equipment failed to produce reliable output at higher frequencies.

**Supplementary references**

Conway BA, Halliday DM, Farmer SF, Shahani U, Maas P (1995) Synchronization between motor cortex and spinal motoneuronal pool during performance of a maintained motor task in man. J Physiol 489.3: 917-924

Friston KJ, Williams S, Howard R, Frackowiak RSJ, Turner R (1996) Movement related effects in fMRI time-series. Mag Res Med 35: 346-355

Glover GH, Li TQ, Ress D (2000) Image-based method for retrospective correction of physiological motion effects in fMRI: RETROICOR. Magnetic Resonance in Medicine 44:162-167

Lund TE; Madsen KH, Sidaros K, Luo W-L, Nichols TE (2006) Non-white noise in fMRI: Does modelling have an impact? NeuroImage 29: 54-66
