# Supplementary material for: Illusory Sensation of Movement Induced by Repetitive Transcranial Magnetic Stimulation
Source: PLoS One. 2010 Oct 11;5(10):e13301. doi: 10.1371/journal.pone.0013301 (PMC2952623; doi:10.1371/journal.pone.0013301)
Supplement: Table S1 — Overview of subject's participation. Age and gender of each subject is written in parenthesis. Subjects marked with X participated in the study. Subject marked with * were lab members and had prior knowledge of the purpose of the experiment, whereas the subjects participating in the main experiment were completely naive towards the hypotheses tested in the experiment. For the SB experiment, subjects marked with ∅ were not included in the analysis (see Methods for details). (0.05 MB DOC) [file pone.0013301.s004.doc]

Subjects included in the study

| **INB subjects** | **Main experiment** | **Control experiment 1** | **Control experiment 2** |
| --- | --- | --- | --- |
| S01 (24y,m) | X |  |  |
| S02 (22y,m) | X |  |  |
| S03 (24y,m) | X |  |  |
| S04 (38y,m) | X |  |  |
| S05 (21y,m) | X |  |  |
| S06 (30y,m) | X |  |  |
| S07 (21y,m) | X |  |  |
| S08 (20y,m) | X |  |  |
| S09 (23y,m) | X | X |  |
| S10 (28y,m) | X | X |  |
| S11*(35y,m) |  | X | X |
| S12*(30y,m) |  | X | X |
| S13*(43y,m) |  | X |  |
| S14*(47y,m) |  |  | X |
| S15*(27y,f) |  |  | X |
| **SB subjects** | **Main experiment** |  |  |
| S16 | Ø |  |  |
| S17 | Ø |  |  |
| S18 (27y,f) | X |  |  |
| S19 (27y,m) | X |  |  |
| S20 (25y,f) | X |  |  |
| S21 | Ø |  |  |
| S22 (35y,m) | X |  |  |
| S23 (27y,m) | X |  |  |
| S24 (27y,f) | X |  |  |
| S25 | Ø |  |  |
